# Pleural metastasis of pulmonary adenocarcinoma mimicking diffuse mesothelioma: A case report and literature study

**DOI:** 10.1016/j.radcr.2022.11.049

**Published:** 2022-12-19

**Authors:** Erriza Shalahuddin, Fierly Hayati

**Affiliations:** Department of Radiology, Faculty of Medicine, Universitas Airlangga, Jl. Mayjed. Prof. dr. Moestopo no 47, Surabaya, East Java, Indonesia

**Keywords:** Pulmonary adenocarcinoma, Mesothelioma, Pleural metastasis

## Abstract

Conditions on pleura cover a broad range of pathology ranging from benign to malignant, which may potentially carry a poor prognosis and lead to high morbidity and mortality. Radiology plays a pivotal role in the diagnosis of pleural malignancy; however, the diagnostic endeavor can be challenging because of overlapping radiological appearances of one condition to another. This case report presents a 61-year-old male with worsening chronic shortness of breath. Despite early imaging resulting in highly suggested mesothelioma, subsequent biopsy proved the malignancy to be pulmonary adenocarcinoma. The patient underwent Pemetrexed-Cisplatin protocol in accordance with the biopsy result, and follow-up imaging depicted a marked improvement of the pleural condition. This case is a prime example of the challenge radiologists have to face regarding pleural tumors and dictates the necessity of a specialized multidisciplinary team to improve the patient's outcome.

## Introduction

Pleural diseases compose a wide range of pathology from benign to malignant, primary or secondary, which may appear with various appearances, whether solitary or diffuse, with multiple involvements. The most common primary malignancy in pleura is mesothelioma, while the metastatic process is the most recurrent secondary malignancy [1]. Both mesothelioma and metastatic process constitute the major proportion of malignancy cases in pleura, with the metastatic process being far more frequent than mesothelioma [2,3]. Mesothelioma is a unique pleural malignancy related to chronic exposure to asbestos and carries an atrocious prognosis [4]. It is reported that the estimated annual incidence of mesothelioma is 10.000 across Western Europe, Scandinavia, North America, Japan, and Australia. Among the said regions, every country reports various incidence rates, from 1.0/100.000 in the United States to 4.2/100.000 in the United Kingdom [1,5].

Radiology plays a pivotal role in the diagnosis of pleural tumors. The chest X-ray is usually the first modality for patients with thoracic complaints [1]. In patients with malignancy suspicion, one of the primary steps is to determine whether the mass is located intrapulmonary, extrapulmonary, or pleural. Several findings suggested that extrapulmonary and pleural origin could be appreciated in chest X-rays, including incomplete border signs and obtuse angle with the thoracic wall [2]. Pleural effusion is the most occurring secondary finding [4] and can be readily visible when a significant volume of fluid is present. Chest CT scan is a cross-sectional imaging modality often used to visualize the lesion further and characterize its relationship with adjacent organs [1]. From a CT scan, radiological appearances of pleural diseases, such as focal or diffuse pleural thickening, pleural plaque, and calcification, can be better depicted. CT findings of particular importance in determining the pleural or extrapleural origin of the mass include erosion of ribs and inward or outward displacement of extrapleural fat [6].

In some exceptional cases, imaging findings of the pleural tumor might be specific. However, in most other cases, the appearance might not be as specific [6]. Moreover, many pleural conditions have overlapping radiological appearances, making diagnostic endeavors even more challenging. Here, in this report, we present a case of diffuse pleural metastasis from lung carcinoma mimicking mesothelioma.

## Case report

A 61-year-old male presented in the outpatient clinic with chronic shortness of breath that had worsened in the past 2 weeks. The patient denied prolonged cough and nocturnal perspiration. He was a carpenter for over 30 years, quit 3 years ago, and he was a non-smoker with no history of cancer. Vital signs were normal; nevertheless, the physical examination revealed apparent respiratory effort with lagging right chest movement. The complete blood test did not indicate a significant abnormality.

**Fig. 1:**
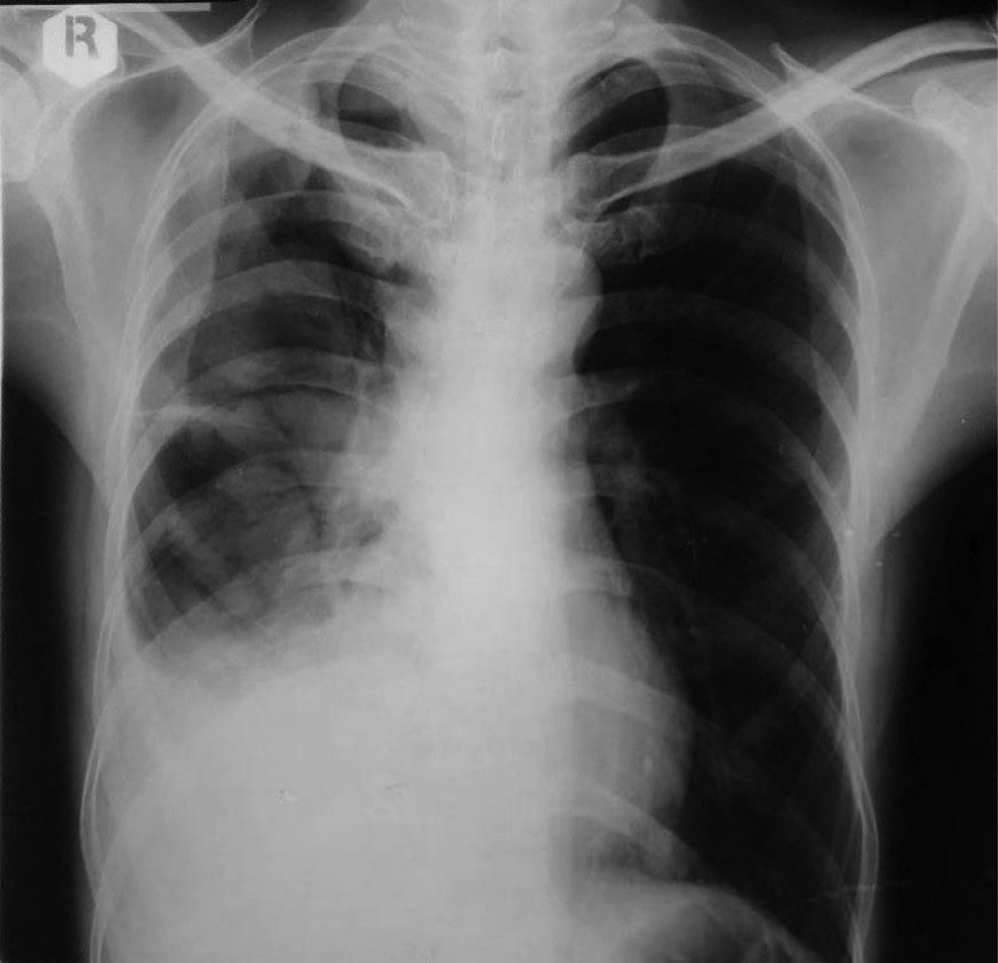
Pleural effusion, fissure thickening, and inhomogeneous opacities on the right lung were seen in the chest X-ray. A nodule was visible in the paracardial site on the left. Left lung hyperaeration was also evident.

Initial chest X-ray (Fig. 1) showed right pleural effusion with multiple heterogeneous opacities on the right lung. A small nodule was identified in the left paracardial with an evident hyperaeration on the left lung.

**Fig. 2:**
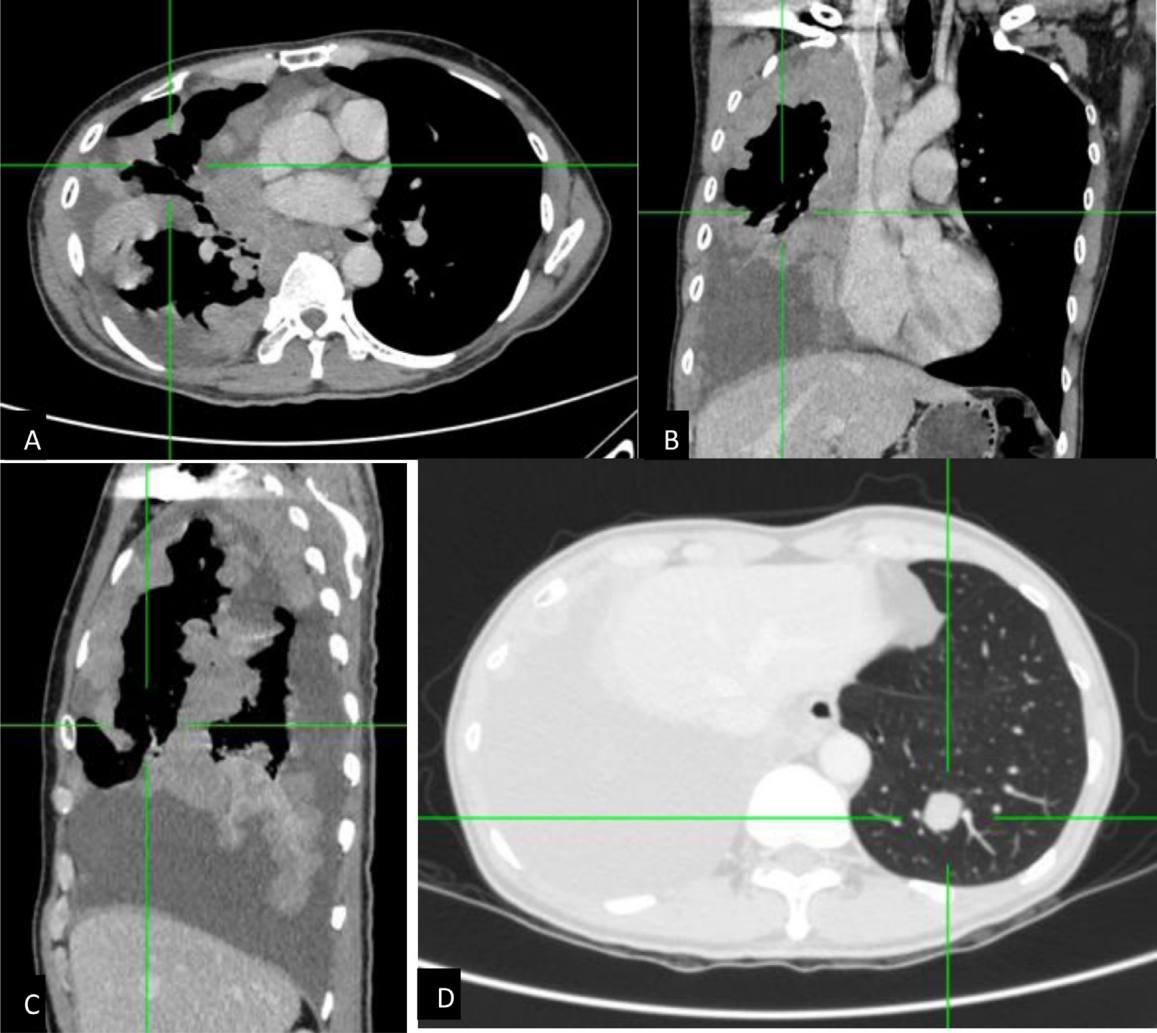
Axial (A) with coronal- (B) and sagittal-reformatted (C) contrast-enhanced chest CT showed diffuse irregular pleural thickening involving fissures, parietal, visceral, mediastinal, and diaphragmatic pleura. Multiple nodules in the left lung were confirmed, with the largest dimension measuring approximately 1.9 cm (D).

Subsequent contrast-enhanced chest CT scan (Fig. 2) demonstrated unilateral diffuse nodular pleural thickening in the right hemithorax involving fissures, parietal, visceral, mediastinal, and diaphragmatic pleura, along with right pleural effusion, causing right compressive atelectasis. No discrete mass was visible in both lung fields, although left lung multiple nodules were noted, and no evidence of chest wall involvement.

**Fig. 3:**
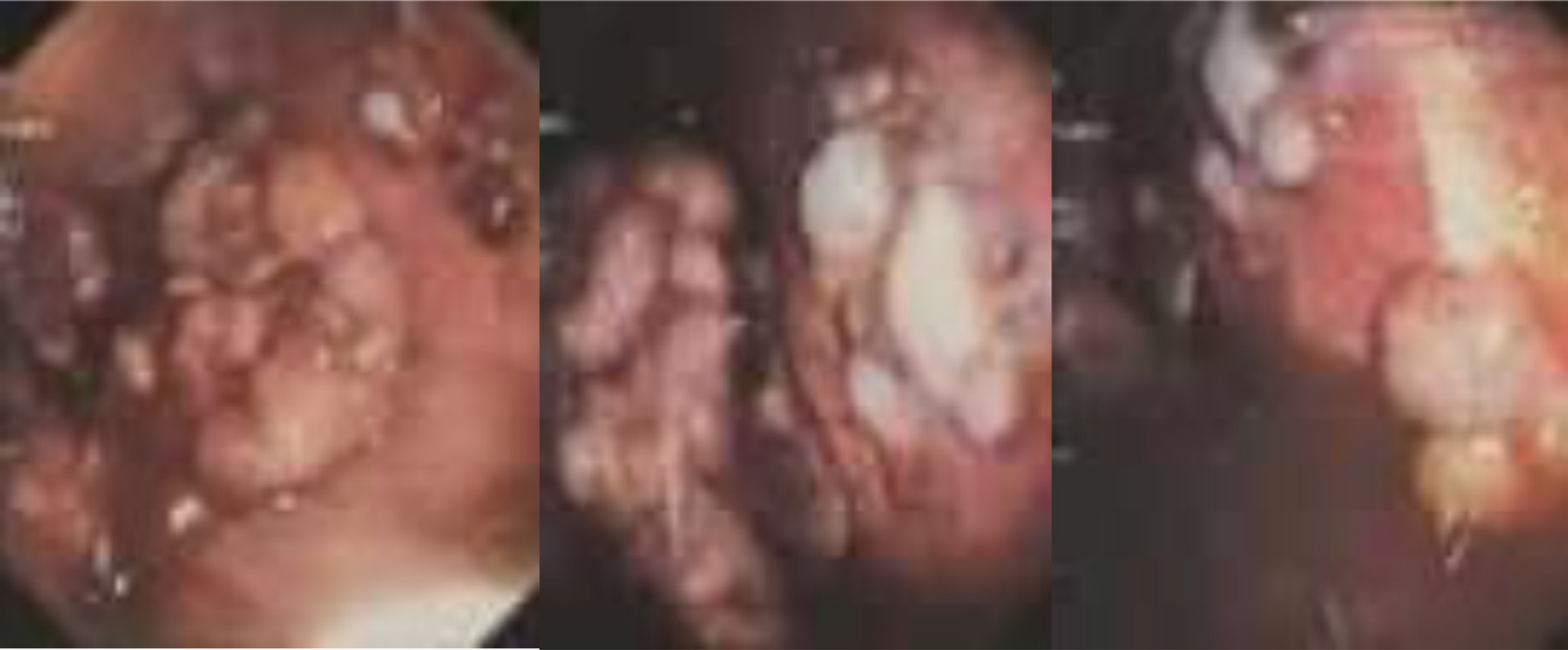
Multiple extensive pleural nodules visualized through thoracoscopy.

**Fig. 4:**
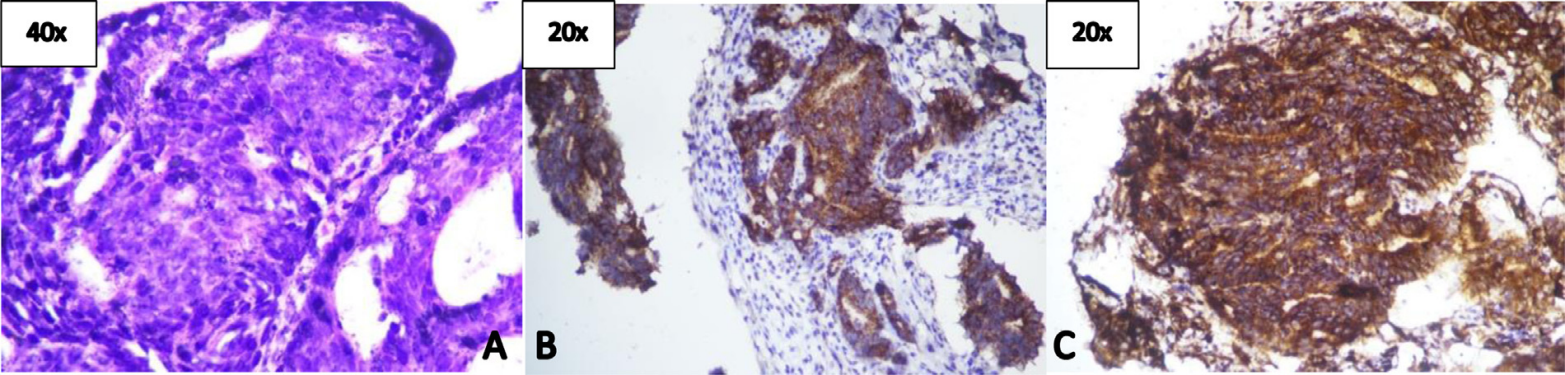
Histopathology from biopsy (A) with 40× magnification showed tumor growth in a glandular pattern, which consists of the proliferation of anaplastic cells with round-oval, pleomorphic, and rough chromatin nuclei, consistent with adenocarcinoma. IHC smear (20× magnification) with Napshin-A positive within the tumor cell cytoplasm (B) and WT-1 negative in tumor cell nuclei (C), suggesting tumor origin from the lung.

No evidence of a distant metastatic process was observed from the bone survey and abdominal ultrasound. Pleural nodules (forceps biopsy, Fig. 3) biopsy and IHC test confirmed pulmonary adenocarcinoma (Fig. 4) with wild-type EGFR mutation in COBAS EGFR test.

**Fig. 5:**
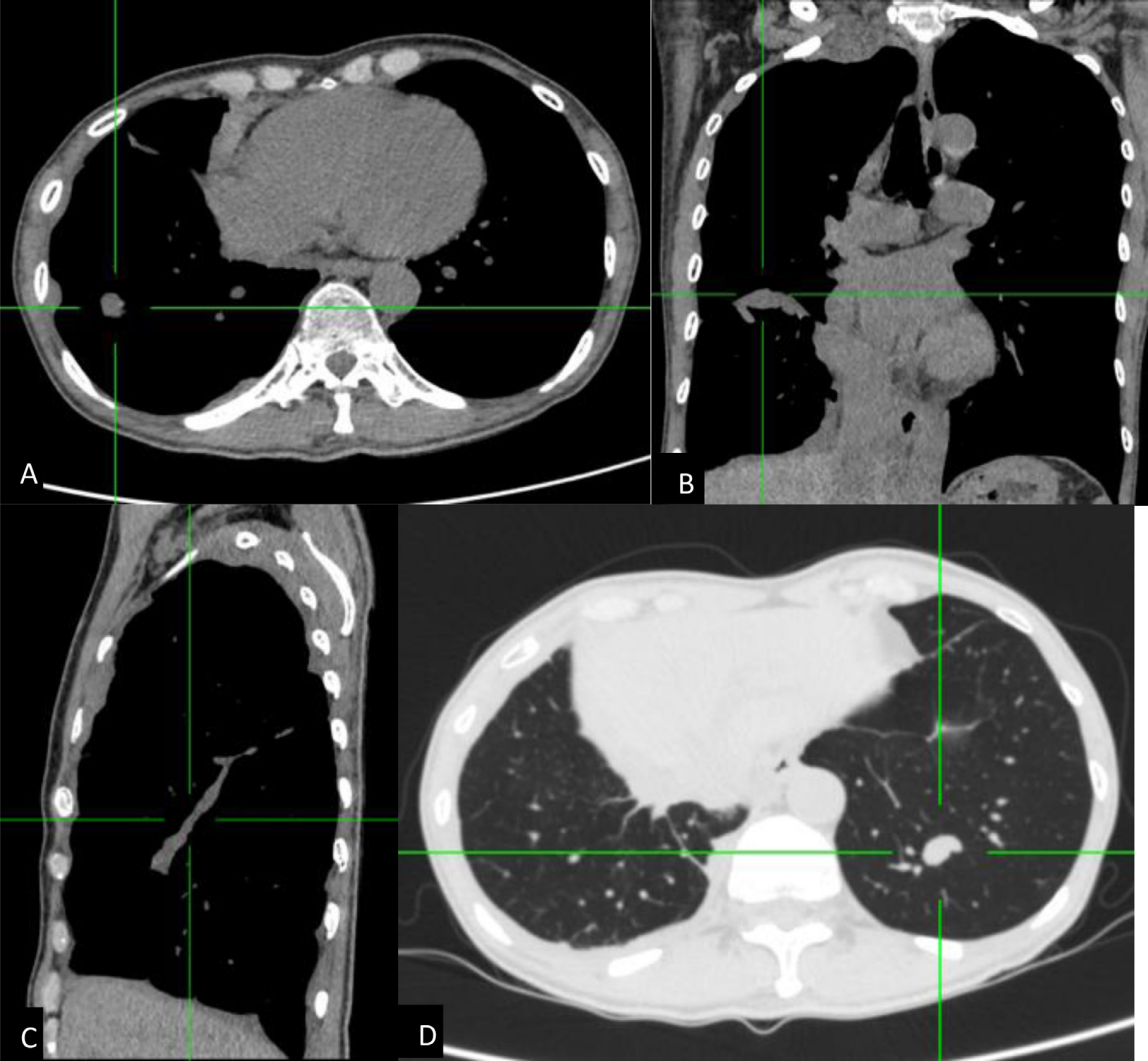
Post-treatment unenhanced chest CT scan in axial (A) with coronal- (B) and sagittal-reformatted (C) plane show marked improvement in overall pleural and fissure thickening. Multiple right lung nodules can be well appreciated with the right lung fully expanded and without right pleural effusion. There is also a reduction in the greatest dimension of the left lung nodule (D), measured at approximately 1.1 cm (greater than 20% reduction).

The patient underwent four cycles of Pemetrexed-Cisplatin protocol according to the biopsy result. Post-treatment CT scan was planned to evaluate treatment results. However, due to elevated blood creatinine serum, only a non-contrast chest CT scan was performed, and this examination was able to note marked improvement of the pleural thickening (Fig. 5).

## Discussion

Our case report raises a challenging question on differentiating between primary pleural malignancy and metastatic process from a radiological standpoint. Our CT findings, with unilateral diffuse nodular pleural thickening, are highly suggestive of mesothelioma. Mesothelioma is known as nodular thickening of the pleura that occur strictly unilateral but circumferentially diffuse, involving fissures and diaphragmatic pleura, and may also affect the pericardium [4,7,8]. Mesothelioma also often causes volume loss in the affected lung, causing elevation of the diaphragm, narrowing of intercostal space, and mediastinal shift [4,7]. Nonetheless, mesothelioma rarely appears as a localized mass [7]. Although common in mesothelioma, pleural effusion is not a specific finding and may occur in other malignant pathologies, such as metastatic processes, and benign processes, such as heart failure [3,8]. In advanced disease, metastatic mediastinal lymph nodes and lung nodules may also present [7].

Histopathology examination in our case, however, has confirmed the pathology to be adenocarcinoma originating from the lung. From our reference study, we found that adenocarcinoma more often metastasizes to pleura than other types of histopathology, with the most common origins from the lung, breast, and ovarium [6]. Radiological findings of pleural metastasis include pleural effusion (most frequently from the breast), as well as focal or diffuse pleural thickening with involvement of parietal and visceral pleura. Lung fissures may also be affected [3]. Although diffuse pleural thickening may be found both in mesothelioma and metastatic process, Kim discovered that circumferential thickening, defined as continuous thickening involving greater than three-quarters of the pleura, is more related to mesothelioma [8]. Pleural metastasis may present as a sizeable solitary mass measuring up to 8-13 cm, as reported in several case reports [9,10].

Adenocarcinoma is a part of the major lung cancer group non-small cell lung cancer (NSCLC), constituting approximately 40% of NSCLC [11]. NSCLC is the most common group of all lung malignancies, constituting 80%-87% of lung cancer [12,13]. The other infrequent group, small cell lung cancer, constitutes the rest and is associated with smoking and is more aggressive [13]. NSCLC may be situated either centrally or peripherally [13], although one study reported that adenocarcinoma has a strong predilection in the peripheral site [12]. Radiological findings may vary from consolidation, ground-glass opacity, or a mix of both [13].

With the absence of discrete mass on visualized lungs, another minor possibility to consider is primary pleural adenocarcinoma. However, in our literature study, we were unable to find any report of primary pleural adenocarcinoma. The closest condition that has been reported is primary pleural squamous cell carcinoma (SCC) [14,15], considering SCC belongs to NSCLC, the same group as adenocarcinoma in lung cancer classification [12]. The reported radiological findings of primary SCC in 2 case reports were single pleural nodule with no signs of invasion [14], bilateral pleural effusion and irregular thickening with involvement of fissures and pericardium, as well as mediastinal lymphadenopathy [15]. Other pleural etiologies considered rare from various literature include pleural lipoma, pleural splenosis, pleural pseudotumor, desmoplastic small round cell tumor, pleural involvement of Erdheim-Chester disease, diffuse pulmonary lymphangiomatosis, synovial sarcoma, solitary fibrous tumor, lymphoma, thymoma, and amyloidosis [1–3,5,6].

Recently, there has been a development of targeted lung cancer therapy due to the discovery of EGFR mutation found in lung cancer. Nevertheless, in our case, the EGFR mutation status was confirmed to be wild-type. Many studies have been conducted to predict the EGFR mutation status of lung cancer based on imaging, stipulating that the EGFR mutation is associated with females and non-smokers. Radiological features associated with EGFR mutation are ground-glass attenuation of the tumor, the presence of air bronchogram, vascular convergence, spiculated margin, and pleural retraction [16]. On the other hand, wild-type EGFR tend to be linked to male and smoking [17] and is less likely to cause diffuse metastatic process [18]. To the best of our knowledge, the study related to EGFR mutation status and metastatic behavior of lung tumors is still relatively limited. EGFR mutation is more likely to cause diffuse "miliary" metastasis involving both lungs [19], whereas EGFR wild-type is more likely to cause pleural metastasis [20]. However, further study is requisite to establish the ground of this concept.

## Conclusion

Pleural mass remains a significant challenge for radiologists. A wide range of pathologies may arise in pleura, and their radiological appearance may overlap one another. A specialized multidisciplinary team is necessary to establish the diagnosis and plan the best course of action for patients' outcomes.

## Ethic committee approval

This study has met the ethical principle and has already obtained approval from Research Ethics Committee at Dr. Soetomo General Hospital, Surabaya.

## Patient consent

Informed consent for patient information to be published in this article was obtained. Appropriate informed consent was obtained for the publication of this case report and accompanying images. This report has been approved by The Ethical Committee of Dr. Soetomo General Academic Hospital.
